# Radical Cure of Oblique Inguinal Hernia by Internal Abdominal Peritoneal Pad

**Published:** 1886-10

**Authors:** William Mac Ewen

**Affiliations:** Glasgow


ON THE RADICAL CURE OF OBLIQUE INGUINAL HERNIA BY INTERNAL ABDOMINAL PERITONEAL PAD, AND THE RESTORATION OF THE VALVED FORM OF THE INGUINAL CANAL. *By* WILLIAM MAC EWEN, M. D., *of Glasgow, in "Annals of Surgery," August, 1886.*

The author's operation is distinguished by his treatment of the sac and method of closing the inguinal canal. After preliminary shaving, disinfection, anæsthesia, and reduction of the hernia, the initial incision is made downward and inward, exposing the external ring. The sac and contents are also explored, the abdominal aspects of internal ring, and position of the epigastric artery. The distal end of the sac is then freed, the sac is pulled down and separated from the cord and the canal, and outside of the sac the peritoneum is detached, with a finger, for half an inch around the whole abdominal aspect of the circumference of the internal ring. A catgut thread, single, and fastened at the distal end of the sac is run in and out several times through the sac, then carried on a hernia-needle through the abdominal wall, except the skin, and brought out at the opening made by the incision. An assistant pulls on this thread, gathering the sac into folds, which form a corrugated pad on the abdominal side of the internal ring. The pad is held in place by traction until the sutures closing the canal are introduced, when the thread is secured. To close the internal ring, an oblique single-thread stitch is taken, with a hernia-needle, in conjoined tendon, from its lower border to the highest point possible. The loop is on peritoneal side. The thread-end, from the lower puncture-point of the stitch, is carried, with a hernia-needle, at a point directly opposite, through the Poupart's ligament and the aponeuroses of the transversalis, and the internal and external oblique. The thread-end from the upper puncture-point, at a point opposite, through the transversalis and the internal and external oblique. The two ends are tied in reef-knot. Stitches may be taken lower down, if necessary, and in uniting the pillars of the external ring care should be taken in tying not to compress the cord. There should be superficial drainage, with permanent dressing, and elastic compression.

In congenital hernia the sac is freed from the canal, opened and divided transversely in two, preserving the cord. The lower part is formed into the *tunica vaginalis*, the upper part is pulled down and split longitudinally for the cord to escape, and is closed by sutures and treated as the sac of acquired hernia. The author prefers catgut for sutures; fourteen to twenty-one day catgut for the deep and for pad, and seven-day catgut for the superficial stitches. Usually but one re-dressing is required. Union is complete in from fourteen to twenty-one days, and the patient is allowed to get up in from four to six weeks. He has thus operated thirty-three times for radical cure of inguinal hernia, in fourteen other cases, after relief of strangulation, and applied the principles in nine cases of femoral hernia, after relief of strangulation. In no case was the operation made where there was a distinct approach to gangrene of the bowel, nor where the sac was firmly adherent next to the femoral vein. There have been no deaths in the series. Firm primary occlusion was obtained in all. There were but few cases of suppuration, and only one was severe. Of the thirty-three, exclusive of two, too recent cases, only one had subsequent need of truss, and that was more from habit. Of the fourteen, three wore a pad and bandage, as precautionary. After the femoral hernias, no truss has been worn.

MARY E. BATES.

